# Licensing effects of inflammatory factors and TLR ligands on the regenerative capacity of adipose-derived mesenchymal stem cells

**DOI:** 10.3389/fcell.2024.1367242

**Published:** 2024-03-28

**Authors:** Diána Szűcs, Tamás Monostori, Vanda Miklós, Zoltán G. Páhi, Szilárd Póliska, Lajos Kemény, Zoltán Veréb

**Affiliations:** ^1^ Regenerative Medicine and Cellular Pharmacology Laboratory, Department of Dermatology and Allergology, University of Szeged, Szeged, Hungary; ^2^ Doctoral School of Clinical Medicine, University of Szeged, Szeged, Hungary; ^3^ Centre of Excellence for Interdisciplinary Research, Development and Innovation, University of Szeged, Szeged, Hungary; ^4^ Biobank, University of Szeged, Szeged, Hungary; ^5^ Genome Integrity and DNA Repair Core Group, Hungarian Centre of Excellence for Molecular Medicine (HCEMM), University of Szeged, Szeged, Hungary; ^6^ Department of Pathology, Albert Szent-Györgyi Medical School, University of Szeged, Szeged, Hungary; ^7^ Genomic Medicine and Bioinformatics Core Facility, Department of Biochemistry and Molecular Biology, Faculty of Medicine, University of Debrecen, Debrecen, Hungary; ^8^ Hungarian Centre of Excellence for Molecular Medicine-USz Skin Research Group, University of Szeged, Szeged, Hungary

**Keywords:** adipose-derived mesenchymal stem cells, regenerative medicine, licensing, inflammation, immune system

## Abstract

**Introduction:** Adipose tissue-derived mesenchymal stem cells are promising contributors to regenerative medicine, exhibiting the ability to regenerate tissues and modulate the immune system, which is particularly beneficial for addressing chronic inflammatory ulcers and wounds. Despite their inherent capabilities, research suggests that pretreatment amplifies therapeutic effectiveness.

**Methods:** Our experimental design exposed adipose-derived mesenchymal stem cells to six inflammatory factors for 24 h. We subsequently evaluated gene expression and proteome profile alterations and observed the wound closure rate post-treatment.

**Results:** Specific pretreatments, such as IL‐1β, notably demonstrated an accelerated wound‐healing process. Analysis of gene and protein expression profiles revealed alterations in pathways associated with tissue regeneration.

**Discussion:** This suggests that licensed cells exhibit potentially higher therapeutic efficiency than untreated cells, shedding light on optimizing regenerative strategies using adipose tissue-derived stem cells.

## 1 Introduction

Adipose tissue is distributed throughout various anatomical sites in the human body, including subcutaneous and visceral locations, intra-articular spaces, intramuscular regions, intra-hepatic depots, and the bone marrow. Beyond an energy reservoir, adipose tissue functions as an endocrine organ, producing many bioactive molecules that modulate metabolic and cellular processes. Among these molecules are adipokines (e.g., leptin, adiponectin, omentin, and resistin), pro- and anti-inflammatory cytokines (e.g., IL-6, TNF-α, IL-1β, IL-8, MCP-1, IL-1Ra, IL-6, IL-7, IL-8, and IL-11), growth factors (e.g., VEGF, HGF, FGF, IGF-1, and BDNF), pro-apoptotic and pro-angiogenic factors, as well as microvesicles enriched with proteins and nucleic acids. Adipose tissue can be categorized into three main types: white adipose tissue, primarily involved in energy storage but also secreting adipokines; brown adipose tissue, responsible for thermogenesis regulation while retaining some energy storage capacity; and beige adipose tissue, which contributes to thermogenesis and energy storage (Konno et al., 2013; Cao et al., 2015; Li and Hua, 2017; Mushahary et al., 2018; Qi et al., 2018; Pittenger et al., 2019; Mazini et al., 2020; Song et al., 2020; Bunnell BA, 2021).

Adipose tissue-derived mesenchymal stem cells (AD-MSCs) reside within adipose tissue, primarily within the stromal vascular fraction (SVF) accessible through minimally invasive procedures. AD-MSCs are multipotent cells characterized by self-renewal potential and the ability to differentiate into mesodermal lineage cells such as adipocytes, chondrocytes, and osteoblasts. They exhibit high proliferation rates and possess immunosuppressive properties, rendering them and their secretome valuable assets in regenerative medicine applications for diseases associated with immune-related disorders. AD-MSCs play a pivotal role in immune response regulation by engaging in direct cell–cell interactions or through the secretion of bioactive factors. These cells interact with various immune cell types, including T cells, B cells, macrophages, natural killer cells (NKs), dendritic cells (DCs), neutrophils, and mast cells (Anton et al., 2012; Konno et al., 2013; Cao et al., 2015; Li and Hua, 2017; Mushahary et al., 2018; Qi et al., 2018; Ridiandries et al., 2018; Zwick et al., 2018; Pittenger et al., 2019; Al-Ghadban and Bunnell, 2020; Mazini et al., 2020; Song et al., 2020; Bunnell BA, 2021; Szucs et al., 2023).

AD-MSCs exert their immunomodulatory influence by interacting with T cells through cell adhesion molecules and modifying the secretion of mediators such as IDO, TGFβ, IL-10, and PGE2. Additionally, T cells reciprocally affect AD-MSCs through chemokine production. Studies have demonstrated that AD-MSCs, in the presence of high pro-inflammatory cytokine levels, promote regulatory T cell (Treg) generation while inhibiting T cell proliferation, activation, and differentiation, thus suppressing immune responses. Conversely, under low pro-inflammatory cytokine exposure conditions, AD-MSCs suppress Treg generation and activate T cell proliferation, activation, and differentiation. AD-MSCs exhibit dual effects on B cells, inhibiting and promoting their proliferation, activation, and differentiation while also inducing chemotaxis and Breg induction. These cells can impede NK cell proliferation, activation, and migration while stimulating NK cell progenitor proliferation and activation.

Furthermore, AD-MSCs hinder DC differentiation, endocytosis, maturation, activation, and migration, inhibiting mast cell degranulation, inflammatory cytokine expression, and chemotaxis. Macrophage polarization is influenced by AD-MSCs, favoring the M2 phenotype and inhibiting the M1 phenotype. At the same time, AD-MSCs modulate neutrophils by inhibiting activation, recruitment, extracellular neutrophil trap formation, and protease secretion while promoting neutrophil survival and recruitment (Anton et al., 2012; Konno et al., 2013; Cao et al., 2015; Li and Hua, 2017; Carelli et al., 2018; Guasti et al., 2018; Mushahary et al., 2018; Qi et al., 2018; Ridiandries et al., 2018; Zwick et al., 2018; Pittenger et al., 2019; Al-Ghadban and Bunnell, 2020; Mazini et al., 2020; Munir et al., 2020; Song et al., 2020; Bunnell BA, 2021; Krampera and Le Blanc, 2021; Szucs et al., 2023).

Due to their immunomodulatory properties, angiogenic potential, and differentiation capacity, AD-MSCs offer a promising avenue for tissue repair (Szucs et al., 2023), regeneration, and replacement in a broad spectrum of conditions characterized by tissue damage. AD-MSCs hold significant therapeutic potential for applications such as wound healing and skin regeneration in various contexts, including diabetic and non-diabetic ulcers, non-healing wounds, extensive burns, and physicochemical skin injuries. Moreover, AD-MSCs find relevance in autoimmune disorders, hematological conditions, graft-versus-host disease, bone and cartilage repair, cardiovascular and muscular diseases, neurodegenerative disorders, and radiation-induced injuries. These versatile cells offer a means to restore tissue function effectively and safely. To ensure secure and successful application in AD-MSC-based therapies, the purity and potency of these cells must be rigorously assessed before administration (Fu et al., 2009; DelaRosa and Lombardo, 2010; Anton et al., 2012; François et al., 2012; Shohara et al., 2012; Konno et al., 2013; Cao et al., 2015; Furuta et al., 2016; Li and Hua, 2017; Carelli et al., 2018; Feldbrin et al., 2018; Guasti et al., 2018; Mushahary et al., 2018; Qi et al., 2018; Ridiandries et al., 2018; Zwick et al., 2018; Pittenger et al., 2019; Sahu et al., 2019; Al-Ghadban and Bunnell, 2020; Kuca-Warnawin et al., 2020; Kurte et al., 2020; Mazini et al., 2020; Munir et al., 2020; Song et al., 2020; Xiao et al., 2020; Bunnell BA, 2021; Krampera and Le Blanc, 2021; Nieto-Nicolau et al., 2021; Krawczenko and Klimczak, 2022; Cheng et al., 2023; Huerta et al., 2023; Szucs et al., 2023).

Adipose tissue-derived mesenchymal stem cells (AD-MSCs) hold significant clinical therapeutic promise attributed to their multifaceted attributes encompassing regenerative, anti-apoptotic, antifibrotic, antioxidant, and immunomodulatory capacities. Beyond the application of AD-MSCs themselves, harnessing their secretome has emerged as an avenue with the potential to influence disease progression positively. Emerging research underscores the dynamic nature of AD-MSC secretion profiles, which can be further tailored through strategic pretreatments, enhancing their suitability for therapeutic applications (Anton et al., 2012; François et al., 2012; Konno et al., 2013; Wu et al., 2013; Cao et al., 2015; Li and Hua, 2017; Guasti et al., 2018; Qi et al., 2018; Zwick et al., 2018; Pittenger et al., 2019; Al-Ghadban and Bunnell, 2020; Mazini et al., 2020; Song et al., 2020; Bunnell BA, 2021; Chang and Nguyen, 2021; Brembilla et al., 2023; Szucs et al., 2023).

Existing literature highlights strategies to augment the effectiveness of MSC-based therapies, focusing on mimicking inflammatory microenvironments. Pro-inflammatory cytokines and hypoxic conditions have been explored as factors capable of potentiating MSC-mediated anti-inflammatory responses. Nevertheless, in these instances, the concomitant detection of classical pro-inflammatory signals has raised questions regarding whether immunosuppressive or pro-inflammatory MSC phenotypes are primarily responsible for the observed therapeutic effects. The complete elucidation of these indications remains a work in progress, with the precise nature of agents and their therapeutically effective concentrations yet to be definitively determined or standardized. Notably, suboptimal conditions may promote the prevalence of a pro-inflammatory MSC phenotype, while excessive concentrations can impact cell viability. In the context of Good Manufacturing Practice (GMP) for cell therapy product manufacturing, pretreatment strategies may also raise regulatory and licensing considerations. However, it is worth highlighting that licensed MSCs often represent the next Frontier in MSC-based therapies, particularly for addressing injuries associated with acute and sub-acute inflammation. Ongoing research continues to delve into the underlying biological processes and the development of safe and efficacious pretreatment approaches. Thus far, the findings have been exceedingly promising, offering significant prospects for advancing the field of regenerative medicine (Anton et al., 2012; François et al., 2012; Konno et al., 2013; Wu et al., 2013; Cao et al., 2015; Li and Hua, 2017; Guasti et al., 2018; Hu and Li, 2018; Qi et al., 2018; Zwick et al., 2018; Pittenger et al., 2019; Al-Ghadban and Bunnell, 2020; Mazini et al., 2020; Song et al., 2020; Bunnell BA, 2021; Chang and Nguyen, 2021; Brembilla et al., 2023; Szucs et al., 2023).

Our study evaluates the wound healing and skin regeneration capabilities of AD-MSCs, particularly in the context of highly inflamed environments, which can influence their expression profile. We hypothesize that the pretreatment of AD-MSCs may be vital to enhancing therapeutic efficacy. Our investigation aims to shed light on the molecular and cellular responses of AD-MSCs to inflammatory factors, specifically LPS, TNFα, IL1β, IFNγ, and PolyI:C. Our findings may contribute to developing more effective therapies where cell preconditioning is pivotal in augmenting therapeutic outcomes. Moreover, our experimental framework has the potential to assess patient-specific responses to inflammatory factors, aiding in the development of personalized therapeutic approaches.

## 2 Materials and methods

### 2.1 AD-MSC isolation

The collection of adipose tissue complied with the guidelines of the Helsinki Declaration, and it was approved by the National Public Health and Medical Officer Service (NPHMOS) and the National Medical Research Council (16821-6/2017/EÜIG, STEM-01/2017), which follows the EU Member States’ Directive 2004/23/EC on presumed written consent practice for tissue collection. Abdominal adipose tissues were removed from patients (Sex:2/3 F/M, Age: 50.2 ± 11.7 years), and the isolation was performed within 1 h after plastic surgery. A detailed description of the AD-MSC isolation protocol can be found in our previous article (Szucs et al., 2023).

### 2.2 Differentiation of adipose-tissue-derived mesenchymal stem cells

The differentiation potential of adipose-tissue-derived mesenchymal stem cells was verified by differentiating into adipocyte, chondrocyte, and osteocyte lines. They were cultured in a 24-well plate, 5 × 10^4^ cells/well; after 24 h of incubation, the differentiation medium was added. The commercially available Gibco’s StemPro^®^ Adipogenesis (A1007001), Osteogenesis (A1007201), and Chondrogenesis (A1007101) Differentiation Kits were applied according to the manufacturer’s guidelines (Gibco, Thermo Fisher Scientific, Waltham, MA United States). After 21 days of maintenance, the cells were fixed with 4% methanol-free formaldehyde (37,308, Molar Chemicals, Hungary) for 20 min at RT. Differentiation stages of AD-MSCs were validated using different dyes. For visualization of lipid-laden particles, Nile red staining (19,123, Sigma-Aldrich, Merck KGaA, Darmstadt, Germany) was utilized, and Alizarin red staining (A5533, Sigma-Aldrich, Merck KGaA, Darmstadt, Germany) was applied to show the mineral deposits during osteogenesis. Toluidine blue staining (89640-5G, Sigma-Aldrich, Merck KGaA, Darmstadt, Germany) was wielded to label the chondrogenic mass.

### 2.3 Flow cytometry

The surface antigen expression pattern was characterized by three-color flow cytometry using fluorochrome-conjugated antibodies with isotype-matching controls. For the measurement of the fluorochrome signal, the BD FACSAriaTM Fusion II flow cytometer (BD Biosciences Immunocytometry Systems, Franklin Lakes, NJ, United States) was applied, and data were processed by Flowing Software (Cell Imaging Core, Turku Centre for Biotechnology, Finland).

### 2.4 Treatment of AD-MSC

The applied AD-MSC cells were derived from the abdominal adipose tissue of three different donors. In a T25 cm^2^ flask, 2.8 × 10^5^ cells were seeded using the upkeeping cell culture media described above, and the cells were incubated for 24 h. Next, the cell culture media was changed for treatment; cells were treated with (A) LPS [100 ng/mL] (tlrl-peklps, ultrapure, Invivogen, San Diego, CA, United States), (B) TNFα [100 ng/mL] (300-01A, Peprotech, London, United Kingdom), (C) IL-1β [10 ng/mL] (200-01B, Peprotech, London, United Kingdom), (D) IFNу [10 ng/mL] (300-02, Peprotech, London, United Kingdom) or (E) PolyI:C [25 ng/mL] (tlrl-pic, Invivogen, San Diego, CA, United States). After adding inflammatory agents, the cells were maintained for 24 h under standard conditions (37°C, 5% CO_2_, untreated cells left as control). Upon 24-h treatment, the cells were collected and processed for RNA isolation.

### 2.5 RNA isolation for RNA-Sequencing

In the context of RNS sequencing, three biological replicates were employed, consistent with the methodology employed in our preceding investigations. The cells were collected, and the pellet was dissolved in 1 mL TRI Reagent^®^ (TR118/200, Genbiotech Argentina, Bueno Aries, Argentina) and kept at −80°C for 24 h. After thawing, 200 µL chloroform (83,627.320, VWR, Radnor, PA, United States) was added to samples, and they were incubated at RT for 10 min after rigorous mixing. For phase separation, the samples were centrifuged at 13,400 g at 4°C for 20 min. The aqueous phase was measured into clean tubes, and 500 µL 2-propanol (SO-9352-B025, Molar Chemicals, Hungary) was added and mixed thoroughly. After this, the incubation and phase-separation steps were repeated. The supernatants were eliminated, and the pellets were washed with 750 µL 75% EtOH-DEPC. The samples were centrifuged at 7,500 *g* at 4°C for 5 min, then the supernatants were discarded, and the samples were dried at 45°C for 20 min. The pellets were suspended in RNase-free water and incubated at 55°C for 10 min. The concentration was measured using an IMPLEN N50 UV/Vis Nanophotometer (Implen GmbH, Munich, Germany), and RNA samples were stored at −80°C until use.

High-throughput mRNA sequencing analysis was implemented on the Illumina sequencing platform to achieve global transcriptome data. The total RNA sample quality was investigated using the Eukaryotic Total RNA Nano Kit according to the manufacturer’s guidelines on Agilent BioAnalyzer. Samples with an RNA integrity number (RIN) value >7 were accepted for the library preparation. RNA-Seq libraries were prepared from total RNA using the Ultra II RNA Sample Prep kit (New England BioLabs) according to the manufacturer’s protocol. In short, poly-A RNAs were captured by oligo-dT conjugated magnetic beads, and then mRNAs were eluted and fragmented at 94°C. First-strand cDNA was created by random priming reverse transcription; then, double-stranded cDNA was made in the second-strand synthesis step. After the reparation of ends, A-tailing and adapter ligation took place. The adapter-ligated fragments were amplified in enrichment PCR, and finally, sequencing libraries were produced. Sequencing runs were performed on the Illumina NextSeq 500 instrument, applying single-end 75-cycle sequencing.

### 2.6 Data analysis

Raw RNA-seq reads were fed into a pipeline to quantify reads mapping to each genomic feature. Quality control (QC) steps were built in at each step of the pipeline, and QC was carried out with FastQC and MultiQC. To remove low-quality bases, short reads, and adapters, Trimmomatic was used. We relied on Illumina’s “Considerations for RNA Seq read length and coverage” (https://knowledge.illumina.com/library-preparation/rna-library-prep/library-preparation-rna-library-prep-reference_material-list/000001243) to determine if the reads were appropriate for further analysis. The next step consisted of aligning the reads to the human genome (GRCh38) using Bowtie2, whereas samples with an alignment percentage over 90% were accepted. This was followed by read quantification using FeatureCounts, a highly efficient general-purpose read summarization program that counts mapped reads for genomic features such as genes, exons, promoters, gene bodies, genomic bins, and chromosomal locations. The resulting count’s table was used for further downstream analysis. For the methods of differential gene expression, PCA analysis, generating the heatmaps of the differentially expressed genes and the pre-selected pathways, and generating volcano plots, see previous article (Szucs et al., 2023). Pathway analysis was conducted using the ViSEAGO package (Brionne et al., 2019). The input for the analysis was a ranked gene list of the differentially expressed genes with *p*-value <0.05 and log2foldchange > |1|. Functional Gene Ontology enrichment was performed with the fgsea package (Korotkevich et al., 2021) using the ViSEAGOrunfgsea command with the “fgseaMultilevel” method (parameters: scoreType = “std”, minSize = 5). Enrichment results were merged (Supplementary Table S1), and semantic similarity measures were made using Wang distance measures, which are based on graph topology. The clustering of the enrichment results was based on the Ward D2 method, and the dendrogram was split into 20 categories (Yu et al., 2010). The heatmap showing these results was made with the ComplexHetamap package (Gu et al., 2016).

### 2.7 Quanterix multi-plex ELISA

For the cytokine assay, we used the Quanterix SP-X digital biomarker analyzer (Quanterix). The system detected simultaneously 10 cytokines in a multiplex assay with Simoa Corplex Cytokine Panel 1 10-Plex Kit (REF: 85-0329, Quanterix). The assay was performed according to the manufacturer’s protocol. Supernatants were thawed, centrifuged, and 4-times diluted with assay diluent. Calibration standards were prepared freshly and measured in triplicates samples in duplicates. After the measurement, results were analyzed and visualized in GraphPad.

### 2.8 *In vitro* scratch assay

AD-MSC cells from three donors were collected and counted using an EVE automatic cell counter (NanoEntek, Hwaseong, Republic of Korea). The *in vitro* scratch assay measures the cell proliferation and migration. The required 1.5×10^4^ cells per well seeded in E-Plate WOUND 96 plates (REF: 300,600,970, Agilent/BioTek, Santa Clara, CA, United States) in maintenance media. The cells were cultured for 24 h under standard conditions (37°C, 5% CO_2_), and then the protocol was separated into two different directions. (I.) Upon 24 h incubation, the scratch was created, and the media was changed immediately to media containing inflammatory agents. It is followed by 48 h of impedance measurement by xCELLigence Real-Time Cell Analyzer (RTCA) device (Agilent/BioTek, Santa Clara, CA, United States). (II.) After 24 h incubation, the media was replaced with media containing inflammatory agents, and the cells were incubated 24 h under standard conditions; then, the scratch was made, and the media was changed immediately to agents-free upkeeping media, followed by 48 h impedance measurement. The scratches were generated using the AccuWound 96 Scratch Tool (Agilent/BioTek, Santa Clara, CA, United States) wound-making device.

### 2.9 RNA isolation and real-time PCR

Upon inflammation-inducing treatments (described above), the Macherey-Nagel NucleoSpin RNA Mini kit (740,955.250, Dueren, Germany) was applied according to the manufacturer’s instructions. All work with RNA and cDNA samples was performed in BioSan UVT-B-AR DNA/RNA UV-Cleaner box (Riga, Latvia). After RNA was extracted and their quality and quantity were verified by IMPLEN N50 UV/Vis nanophotometer, the cDNA synthesis was performed using High-Capacity cDNA Reverse Transcription Kit (4,368,813, Applied Biosystems™, Thermo Fisher Scientific, Waltham, MA, United States) according to the protocol of the manufacturer. Analytik Jena qTOWER^3^ G Touch Real-Time Thermal Cycler (Jena, Germany) was utilized for reverse transcription.

### 2.10 qPCR

The Xceed qPCR Probe 2x Mix No-ROX kit (NPCR10502L, Institute of Applied Biotechnologies, Prague, Czech Republic) and TaqMan probes (4,331,182, 250 rxns, FAM-MGB, Thermo Fisher Scientific, Waltham, MA, United States) were used for quantitative PCR. Three biological and three technical replicates were applied in all cases, and data were analyzed by 2^−ΔΔCT^ method. The protocol was executed according to the manufacturer’s instructions.

### 2.11 ELISA

For measurement of cytokine/chemokine levels in cells upon inflammation induction, the Human IL-6 ELISA set (555220), Human IL-8 ELISA set (555244), Human IP-10 ELISA set (550926), and Reagent Set B (550534) were applied from BD OptEIA™ (BD Biosciences, Franklin Lakes, NJ, United States) according to the guidelines of the manufacturer.

### 2.12 Cytotoxic effect of inflammatory agents on AD-MSCs

To measure the cytotoxicity of the given treatment, the Cytotoxicity Detection Kit (LDH) (REF: 11644793001, Roche, Basel, Switzerland) was applied according to the manufacturer’s instructions. Cell supernatants were collected upon 24 h of inflammation induction (described above), and the colorimetric absorbance measurement was executed by Synergy HTX multiplate reader (Agilent/BioTek, Santa Clara, CA, United States) on 490 nm, reference wavelength was 620 nm.

### 2.13 Cell proliferation and metabolism

Effects on proliferation were measured with BrdU Cell Proliferation ELISA Kit (Ref: 11647229001, Roche, Basel, Switzerland). The thymidine analog 5-bromo-2′-deoxyuridine (BrdU) is incorporated into the DNA, thus allowing the determination of the inhibitory or stimulatory effect on the proliferation of the activator molecules. 1,5 × 10^4^ cells per well were seeded into a 96-well plate. The inflammatory environment was simulated with the materials and concentrations mentioned above. The treatment lasted 24 h, and the assays were performed according to the manufacturer’s protocol. Measurement was performed on Synergy HTX multiplate reader (Agilent/BioTek, Santa Clara, CA, United States) at 550 nm, with reference at 650 nm for MTT assay, on 450 nm, with reference at 690 nm.

Changes in metabolism due to an inflammatory environment were examined by Cell Proliferation Kit (MTT) (Ref: 11465007001, Roche, Basel, Switzerland) according to the manufacturer’s guidelines. The bioreduction of tetrazolium salt to formazan gives information about cellular metabolism and viability. 1.5 × 10^4^ cells per well were seeded into a 96-well plate, then the cells were incubated for 24 h under standard conditions (37°C, 5% CO_2_). Next, the 24 h induction of inflammation was taken place (described above), and the colorimetric absorbance measurement was carried out by Synergy HTX multiplate reader (Agilent/BioTek, Santa Clara, CA, United States) on 550 nm, reference wavelength was 650 nm.

## 3 Results

### 3.1 Overall transcriptomic profile of treated cells

**FIGURE 1:**
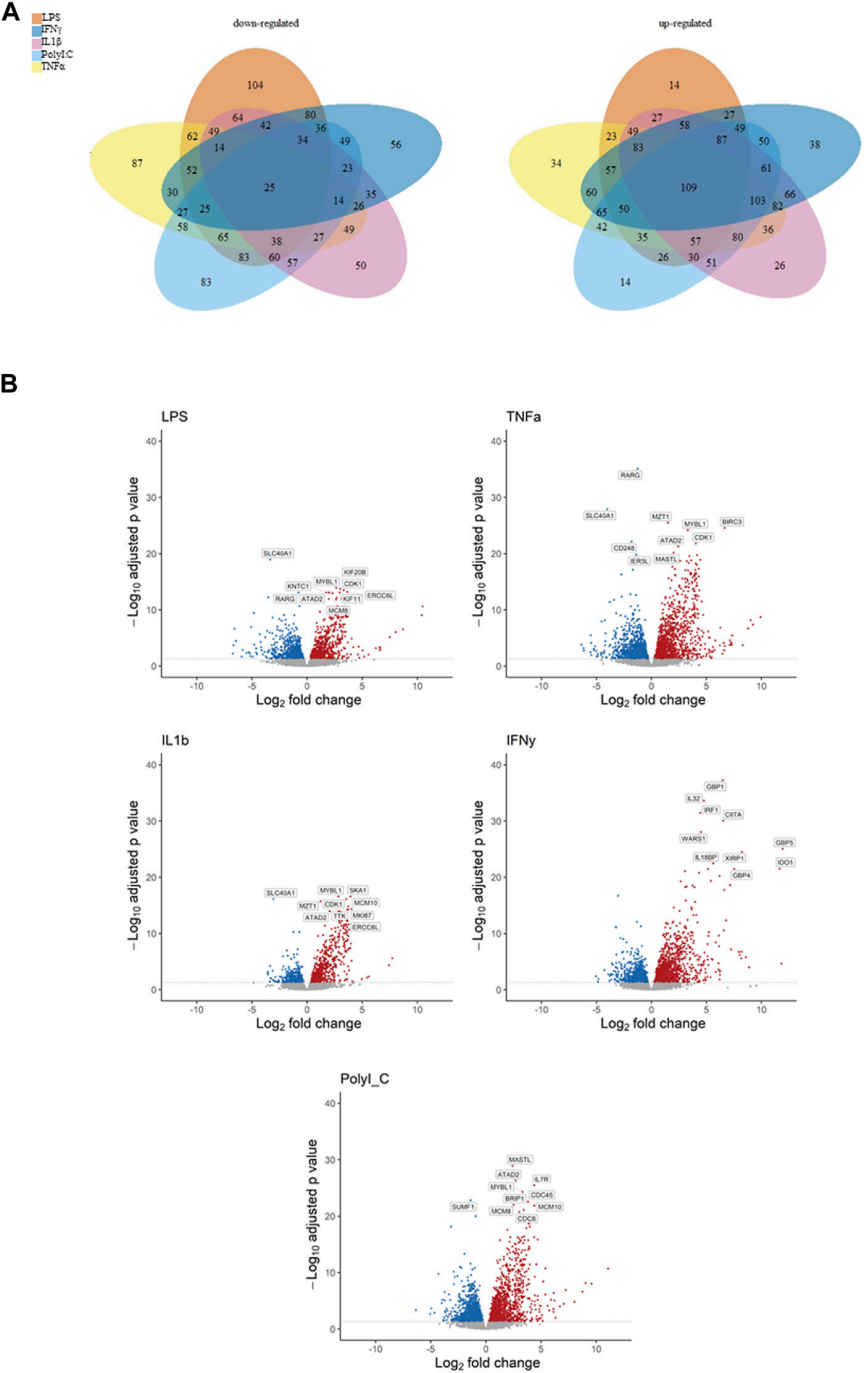
Representation of down- and upregulated DEGs after pro-inflammatory treatment compared to the control condition, **(A)** Venn diagrams illustrating overlapping gene numbers between the treatments, and Volcano plots **(B)** depicting the most significantly altered genes, where p adjusted < 0.05 are shown in red, significantly downregulated genes p adjusted < 0.05 are shown in blue.

The Venn diagrams illustrate the extensive impact of the treatments on gene expression, revealing both upregulation and downregulation of numerous genes, along with significant intersections (Figure 1A). Specifically, the treatments exhibited downregulation of genes as follows: LPS (104 genes), TNF-α (87 genes), IL-1β (50 genes), IFN-γ (56 genes), and PolyI:C (83 genes), with a collective influence on 25 shared genes. Conversely, the treatments led to the upregulation of multiple genes: LPS (14 genes), TNF-α (34 genes), IL-1β (26 genes), IFN-γ (38 genes), and PolyI:C (14 genes), with a common effect observed in 109 genes. These findings underscore the intricate and multifaceted impact of the treatments on the transcriptomic profile, revealing both shared and unique regulatory responses across the treated conditions. The Volcano plot illustrates extensive alterations in gene expression induced by the treatments, encompassing up and downregulation of genes’ expression (Figure 1B). Notably, among the top 10 most significantly altered genes, a majority exhibition.

### 3.2 ViSEAGO and clustered pathways

**FIGURE 2:**
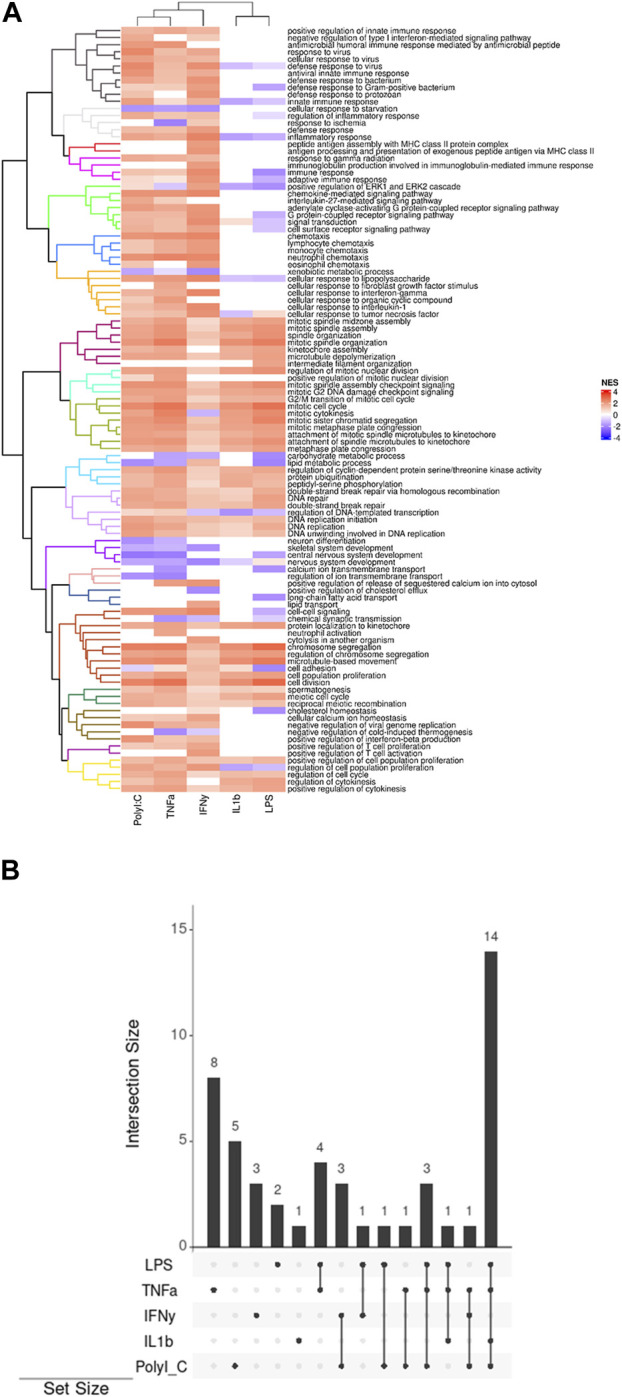
**(A)** Display of clustered pathways heatmap generated by ViSEAGO. The different colors represent uniform groups, including several pathways and biological processes **(B)** The UpSet plots visually represent the gene intersections within various pathways in the AD-MSC following treatment with pro-inflammatory agents. The vertical bars highlight the number of shared genes, denoting the intersection size, among specific pathway sets, indicated by bottom-filled connected circles.

The heatmap generated through ViSEAGO analysis delineates treatments into two primary clusters, with PolyI:C, TNF-α, and IFN-γ forming one cluster and IL-1β and LPS constituting the other (Figure 2A). There is a difference between the two groups in defense and immune response, signal transduction, chemotaxis, cellular response to chemical stimulus, biological regulation, and T-cell activation. They have common effects on organelle organization, mitotic cell cycle, primary metabolic process, DNA repair, system development, ion transmembrane transport, lipid transport, cellular process, meiotic cell cycle, and cell cycle regulation.

The treatments exert distinct effects on multiple pathways: LPS impacts two pathways, TNFα influences eight pathways, IL-1β affects one pathway, IFN-γ modulates three pathways, and PolyI:C impacts five pathways (Figure 2B). This differential influence on various molecular pathways underscores the complexity of the treatments’ interactions with cellular processes. The treatments also elicit shared alterations in multiple pathways. Specifically, LPS and TNFα affect four common pathways, while IFNγ and PolyI:C induce changes in three overlapping pathways. Additionally, LPS and IFN-γ influence one common pathway, as do LPS and PolyI:C, and TNFα and PolyI:C. Notably, the combined treatment of LPS, TNFα, and PolyI:C results in concurrent modifications in three pathways, whereas the combination of LPS, TNFα, and IL-1β influences one shared pathway. Furthermore, TNFα, IFNγ, and PolyI:C collectively impact one common pathway. Lastly, the joint influence of LPS, TNFα, IL-1β, and PolyI:C is reflected in alterations in 14 pathways, emphasizing the intricate interplay of these treatments in affecting cellular processes.

**FIGURE 3:**
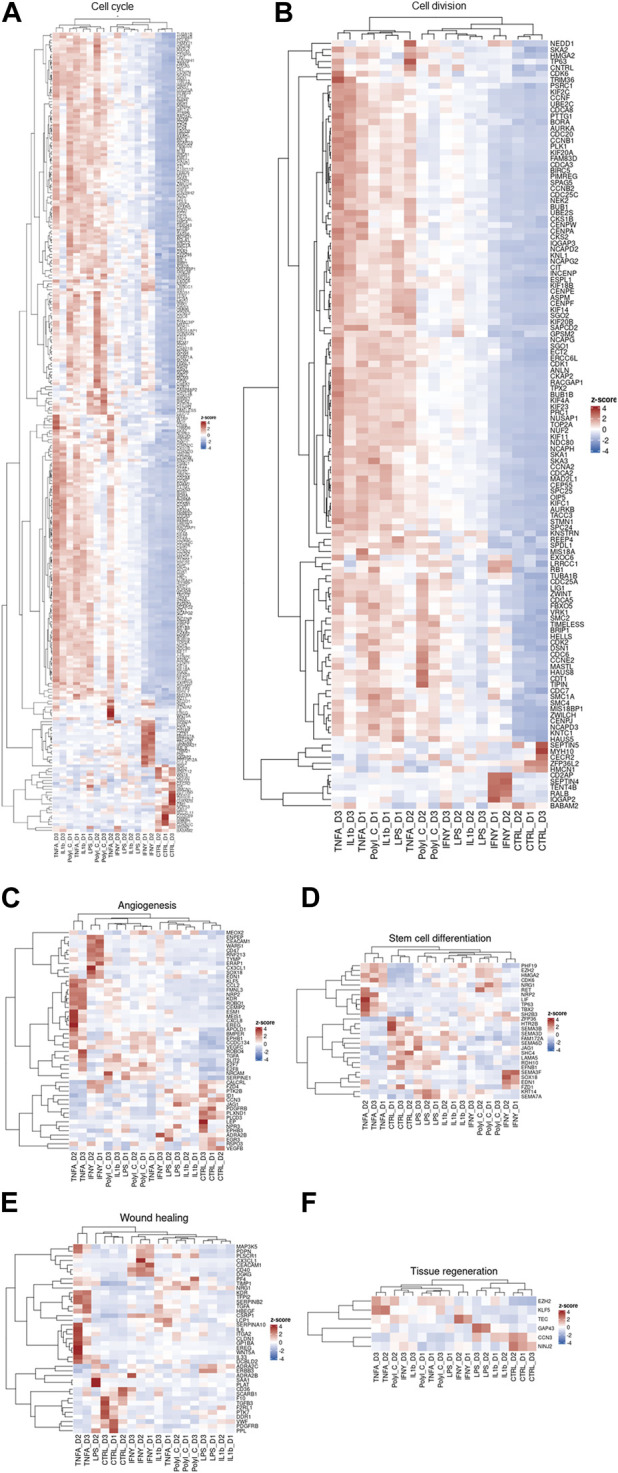
Clustered heatmaps depicting various processes: **(A)** Cell cycle, **(B)** Cell division, **(C)** Angiogenesis, **(D)** Stem cell differentiation, **(E)** Wound healing, and **(F)** Tissue regeneration. These biological processes are crucial for AD-MSC tissue regeneration, healing, and immunomodulation of the microenvironment.

Our transcriptomic dataset has been visualized through a series of heatmaps, categorized based on predefined pathways (Figure 3). Across all heatmaps, the distinction is evident between the transcriptional patterns of treated and control samples, albeit with notable inter-donor variations. This dataset is an illustrative exemplar of the diverse cellular responses induced by treatments, each reflecting the unique molecular landscape of individual patients. Notably, genes modulated by TNF-α and IFN-γ in the angiogenesis pathway heatmap tend to cluster together. In the context of cell cycle and cell division pathways, TNF-α, IL-1β, and PolyI:C appear to share analogous effects, whereas IFN-γ delineates a distinct grouping. Regarding stem cell differentiation, the impact of LPS and IL-1β aligns closely with that of the control group. TNF-α exhibits similarity with PolyI:C and IFN-γ manifests as an independent cluster.

Furthermore, the heatmap of tissue regeneration reveals that LPS and IL-1β treatments display comparable profiles to the control group, whereas TNF-α delineates discrete gene clusters. LPS and IL-1β treatments diverge from the control group in wound healing pathways, whereas TNF-α, IFN-γ, and PolyI:C treatments segregate into distinct clusters. These observations highlight the nuanced and pathway-specific effects of the treatments on cellular responses, underlining the complexity of treatment outcomes across diverse biological contexts.

### 3.3 Perform cellular characterization and evaluate the safety of treatment

**FIGURE 4:**
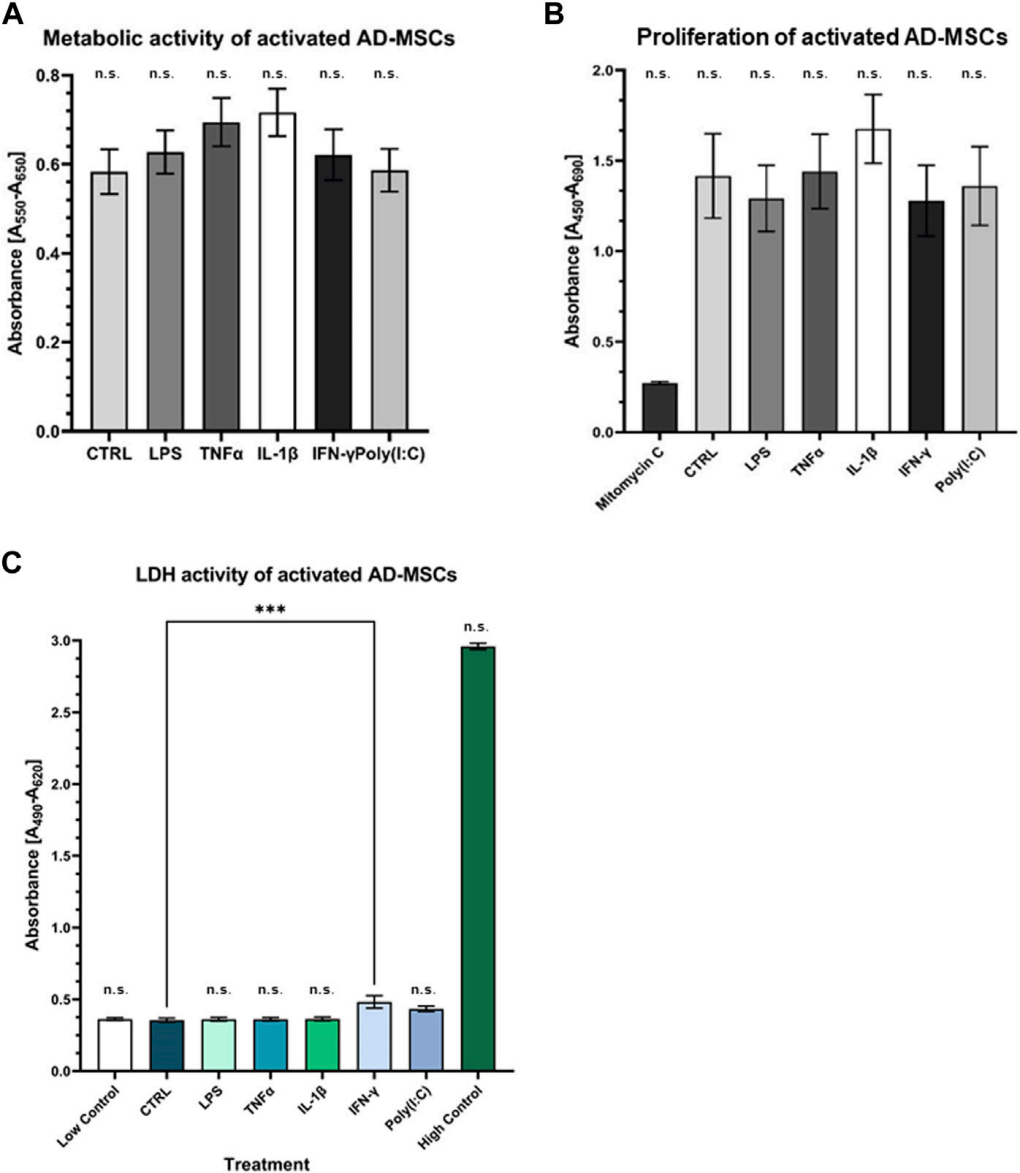
Assessment of metabolic and proliferation activity of treated AD-MSCs **(A)** The metabolic activity of the treated cells was detected by MTT assay. There is no significant change compared to the control. **(B)** BrdU incorporation indicated the proliferative capacity of cells, which was not significantly affected by treatments. **(C)** Cell viability was not affected by any treatments, indicating that our results were not caused by dead cells. (N = 3 donors, each measurement performed in triplicates).

Primary cell cultures were maintained, and their differentiation potential was assessed. Remarkably, these cells demonstrated tri-lineage differentiation capacity, encompassing adipogenic, chondrogenic, and osteogenic lineages (Supplementary Figure S1). This differentiation was verified through microscopic examination of distinct cell type-specific features, reinforcing the suitability of these cultures as a valuable model for our study. The FACS analysis revealed crucial cell characteristics and verified their mesenchymal origin, bolstering their identity and facilitating further exploration of their unique traits and functions in our study [data not shown; see previous article by (12)]. Cytotoxicity and viability tests showed that the cells did not receive a cytotoxic amount of inflammatory agents, and their metabolism and viability were preserved during treatment, underscoring the safety and potential therapeutic relevance of the administered agents in cellular health and function (Figure 4).

### 3.4 Influence of treatments on the cell proliferation and migration

**FIGURE 5:**
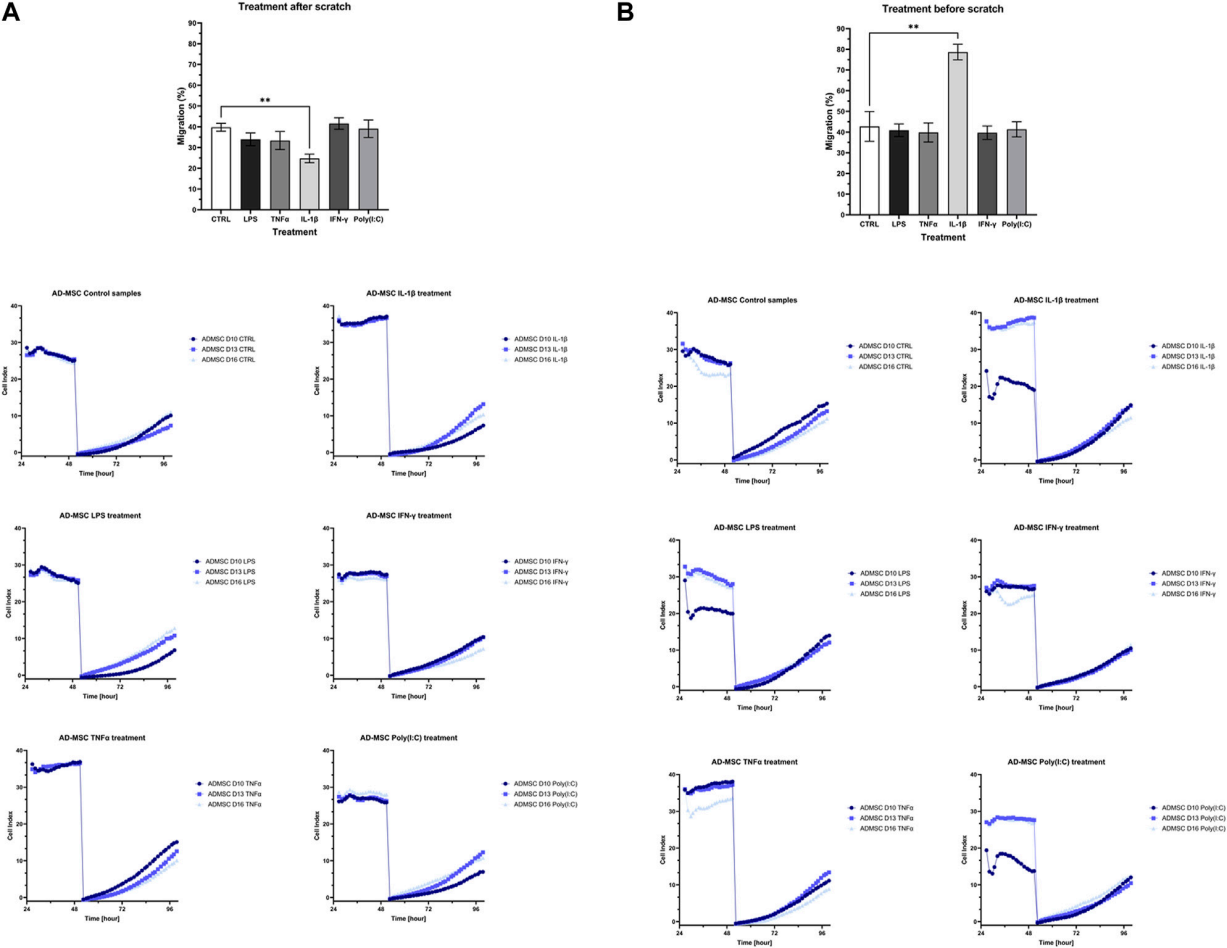
The bar charts and diagrams show the results of the cell proliferation and migration studies with two visualized layouts. The impedance-based method measured the re-population of the direct wound size. **(A)** “treatment before scratch”, which is intended to model that the wound is initially in an inflammatory environment, and **(B)** “treatment after scratch”, which is designed to model an infected wound. Both of these cases have a clinical manifestation in chronic non-healing wounds and, e.g., infectious wounds associated with diabetes.

The cell proliferation and migration assay utilized impedance measurements to assess the rate of cell migration during wound closure in treated samples relative to a control group after the injury. Two experimental conditions were examined: one where the wound was introduced before treatment (Figure 5A) and another where treatment preceded wound induction (Figure 5B). In both cases, distinct variations in wound closure dynamics were evident between cells subjected to IL-1β treatment and the control group. Specifically, when applied before wound initiation, IL-1β significantly hastened wound closure, whereas its post-wound application decelerated the process.

### 3.5 Gene and protein expression analysis by qPCR and ELISA

**FIGURE 6:**
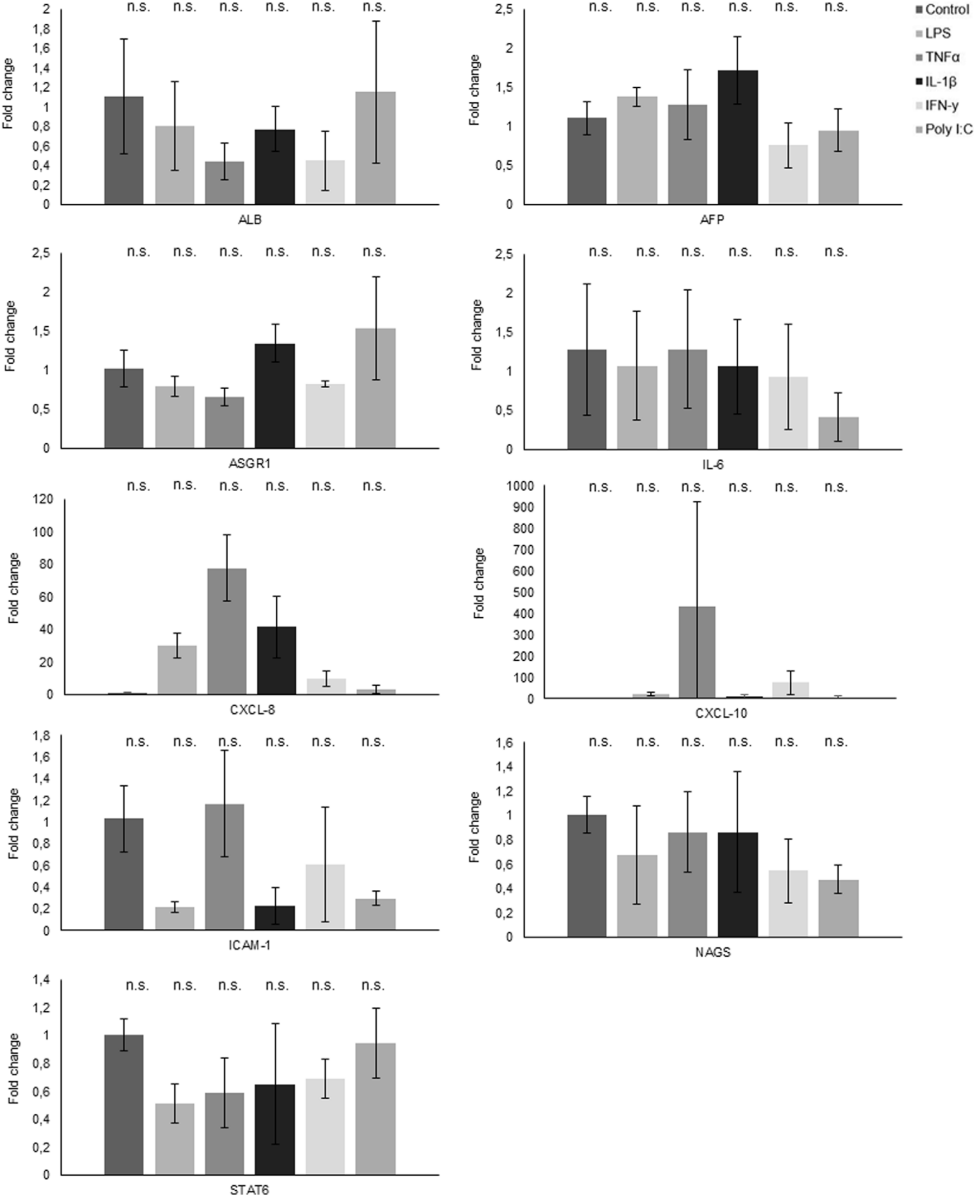
Gene expression analysis through qPCR reveals the fold change in various genes following treatments.

The quantitative polymerase chain reaction (qPCR) analysis results unveiled distinct gene expression patterns in response to various treatments (Figure 6). Notably, CXCL-8 demonstrated a robust upregulation in response to all treatments, with a marked increase following exposure to LPS, TNFα, and IL-1β. Conversely, both NAGS and STAT6 exhibited consistent downregulation across all treatment conditions. Interestingly, while TNFα treatment had no appreciable effect on IL-6 expression, the other administered treatments induced a significant reduction in IL-6 mRNA levels.

Furthermore, CXCL-10 displayed an elevated expression profile in response to TNFα treatment and a modest increase following IFNγ exposure. Conversely, ASGR1 exhibited a notable decrease in expression levels following treatment with LPS, TNFα, and IFNγ, while it demonstrated an elevation in response to IL1β and PolyI:C. In the case of ICAM1, its expression slightly increased following TNFα treatment, but conversely, it experienced a decrease when subjected to all other treatments.

**FIGURE 7:**
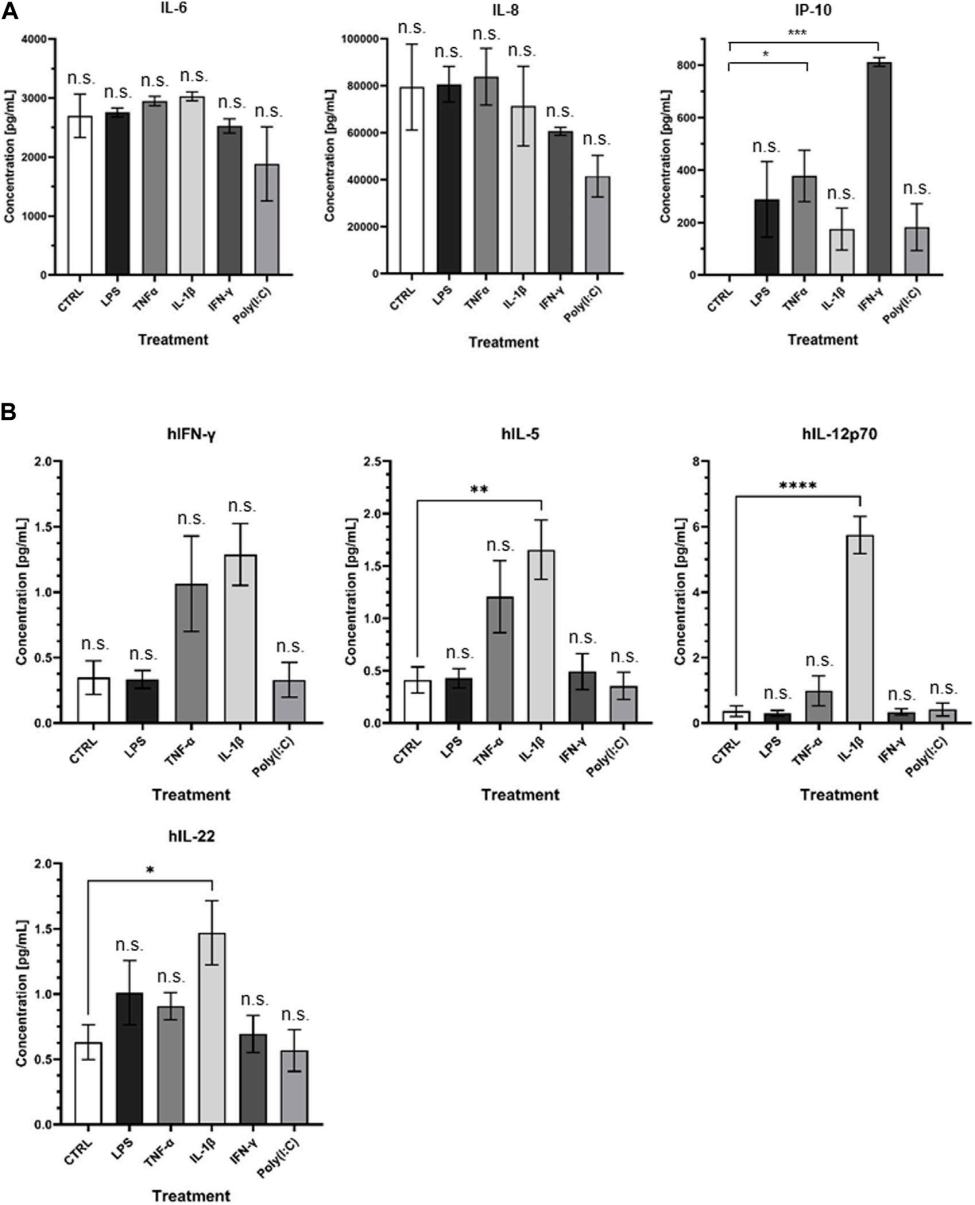
**(A)** Visual representation of ELISA results and **(B)** presentation of Quanterix findings.

At the protein level, noteworthy distinctions emerge (Figure 7A). Specifically, the treatments with LPS, TNFα, and IL-1β resulted in a notable upsurge in IL-6 levels, whereas IFNγ and Poly I:C treatments led to a discernible reduction in IL-6 protein concentrations. CXCL-8 exhibited an augmentation in response to LPS and TNFα treatments, contrasted by a diminishment observed following the remaining treatment regimens. Interestingly, CXCL-10 demonstrated an elevation in protein levels across all administered treatments, with particularly significant peaks observed following TNFα and IFNγ treatments. The multiplex ELISA findings reveal notable changes in the IFNγ, IL-5, IL-12p70, and IL-22 levels. Specifically, IL-5, IL-12p70, and IL-22 significantly increased following IL-1β treatment (Figure 7B).

## 4 Discussion

Mesenchymal stem cells (MSCs) are crucial in various immunological processes. They actively regulate their microenvironment and influence the differentiation of different cells, including immune cells, by producing cytokines and growth factors (Saparov et al., 2016). In their basal state, MSCs exhibit antiangiogenic properties. The immunomodulatory effectiveness of MSCs is contingent upon the nature and intensity of inflammatory signals received, such as interferon-γ (IFN-γ), tumor necrosis factor-α (TNF-α), and Toll-like receptor (TLR)-mediated activation. Under specific inflammatory stimuli (IFN-γ, TNF-α, TLR-mediated activation), MSCs transform, becoming antiapoptotic, proangiogenic, and immunosuppressive (Meisel et al., 2004; Krampera et al., 2006; Ghannam et al., 2010; Saparov et al., 2016; Vereb et al., 2020). They contribute to inflammation reduction through the secretion of factors like interleukin-6 (IL-6), indoleamine 2,3-dioxygenase (IDO), HLA G5 (human leukocyte antigen G5), interleukin-10 (IL-10), transforming growth factor beta-1 (TGFb1), hepatocyte growth factor (HGF), HOX-1, IL-1Ra (IL-1 receptor antagonist), prostaglandin E2 (PGE2), and through cell-cell contact (Bartholomew et al., 2002; Meisel et al., 2004; Aggarwal SP and Pittenger, 2005; Saparov et al., 2016). The immunomodulatory potential of MSCs thus hinges on their response to specific inflammatory cues (Renner et al., 2009; Li et al., 2012). Mesenchymal stem cells (MSCs) actively produce a diverse array of chemokines and adhesion molecules, including ligands for CXC chemokine receptor 3 (CXCR3), C-C chemokine receptor type 5 (CCR5), intercellular adhesion molecule 1 (ICAM-1/CD54), and vascular cell adhesion molecule 1 (VCAM-1) (Vereb et al., 2016; Vereb et al., 2020). The pronounced expression of CXCR3 in effector and memory T cells underscores the pivotal significance of MSC-generated chemokines, particularly CXCL9 (chemokine ligand 9), CXCL10 (chemokine ligand 10), and CXCL11 (chemokine ligand 11). These chemokines play a critical role in orchestrating the recruitment of lymphocytes to the site of tissue damage, thereby ensuring optimal functionality of immunosuppression (Crop et al., 2010; Wang et al., 2014; Saparov et al., 2016). The ability of MSCs to influence the immune system and the effects are intricately tied to variables such as the tissue origin (whether from fat, bone marrow, *etc.*), the specific microenvironment they inhabit, and the nature of their interactions with other cellular partners. The role of adipose-derived mesenchymal stem cells (AD-MSCs) in wound healing is a subject of considerable significance. These cells exhibit a wide array of regenerative, anti-apoptotic, antifibrotic, anti-oxidative, and immunomodulatory properties, rendering them invaluable for therapeutic applications, particularly in wound healing (Neuss et al., 2004; Fu et al., 2009; DelaRosa and Lombardo, 2010; Trayhurn et al., 2011; Shohara et al., 2012; Konno et al., 2013; Cao et al., 2015; Ti et al., 2015; Furuta et al., 2016; Carelli et al., 2018; Feldbrin et al., 2018; Mushahary et al., 2018; Ridiandries et al., 2018; Sahu et al., 2019; Al-Ghadban and Bunnell, 2020; Kuca-Warnawin et al., 2020; Kurte et al., 2020; Munir et al., 2020; Xiao et al., 2020; Bunnell BA, 2021; Krampera and Le Blanc, 2021; Nieto-Nicolau et al., 2021; Krawczenko and Klimczak, 2022; Skibber et al., 2022; Wiese et al., 2022; Cheng et al., 2023; Huerta et al., 2023).

Moreover, the secretome of AD-MSCs, the array of substy release, has positively affected various diseases. Research has focused on the impact of priming or pre-conditioning AD-MSCs with pro-inflammatory cytokines, such as interferon-gamma (IFN-γ) and tumor necrosis factor-alpha (TNFα), on their immunomodulatory capabilities. This treatment enhances their potential to suppress the immune response by upregulating specific genes associated with signaling proteins, immune molecules, and cell surface markers (Heo et al., 2011; Konno et al., 2013; Cao et al., 2015; Saparov et al., 2016; Chinnadurai et al., 2017; Carvalho et al., 2019; Al-Ghadban and Bunnell, 2020; Bunnell BA, 2021). However, the precise balance between immunosuppressive and pro-inflammatory effects remains an area of ongoing exploration. Mesenchymal stem cell (MSC) therapy has experienced substantial growth over the last two decades, with over 1,000 trials conducted.

Nevertheless, only a tiny fraction has progressed to industry-sponsored phase III trials, primarily due to the relative novelty of this field. Challenges persist in optimizing cell quantity delivery methods and comprehending the importance of MSC localization at the injury site. Licensing AD-MSCs with IFN-γ is suggested to enhance their immunomodulatory potential, with clinical experiences showing potential for treating immune-related diseases (Waterman et al., 2010; Heo et al., 2011; Konno et al., 2013; Cao et al., 2015; Saparov et al., 2016; Chinnadurai et al., 2017; Hu and Li, 2018; Carvalho et al., 2019; Al-Ghadban and Bunnell, 2020; Bunnell BA, 2021; Lu and Qiao, 2021). Immunoglobulin kappa chains in various cancer cell types have also garnered attention in recent studies (Chen et al., 2009; Zhao et al., 2021). These investigations underscore the significance of key proteins like RAG1, RAG2, and AID, which are pivotal for immunoglobulin production and rearrangement (Zhao et al., 2021). Although emerging evidence suggests a potential role of immunoglobulin expression in promoting cancer cell growth, the functional consequences remain unclear. Given the vital roles of immunoglobulins in human physiology and disease management, in-depth research is crucial to uncover their multifaceted functions, particularly in cancer etiology and therapeutic strategies. In conclusion, these studies collectively emphasize the importance of AD-MSCs in wound healing, their licensing with pro-inflammatory cytokines, and the intriguing role of immunoglobulins in various cellular contexts, particularly in cancer. Ongoing research holds promise for further advancements in regenerative medicine and cancer biology (Chen et al., 2009; Zhao et al., 2021).

The immense potential of adipose-derived mesenchymal stem cells (AD-MSCs) in regenerative medicine is undeniable. Their regenerative, anti-apoptotic, antifibrotic, anti-oxidative, and immunomodulatory qualities offer substantial promise for clinical therapy (DelaRosa and Lombardo, 2010; Konno et al., 2013; Cao et al., 2015; Carelli et al., 2018; Qi et al., 2018; Pittenger et al., 2019; Song et al., 2020; Bunnell BA, 2021; Szucs et al., 2023). Additionally, our evolving comprehension of AD-MSCs has unveiled the dynamic role played by their secretome and the substances they release (Trayhurn et al., 2011). This secretome can be customized through specific pretreatments, enhancing its therapeutic adaptability. Investigations into the paracrine effects of AD-MSCs have yielded compelling findings. For instance, pre-conditioning AD-MSCs with tumor necrosis factor-alpha (TNFα) and applying their secretome to cutaneous wounds in rats has remarkably expedited wound healing, proliferation, angiogenesis, epithelialization, and macrophage recruitment (Heo et al., 2011; Saparov et al., 2016). Notably, pro-inflammatory cytokines like IL-6 and IL-8 have been recognized as contributors to this acceleration (Saparov et al., 2016; Heo et al., 2011) Similarly, AD-MSCs subjected to pre-treatment with lipopolysaccharides (LPS) have demonstrated their potential in promoting wound healing and angiogenesis, coupled with an increased release of growth factors associated with tissue regeneration and immune responses (Wang et al., 2022). AD-MSCs have showcased their immunomodulatory capabilities in a mouse model of atopic dermatitis, effectively suppressing B-lymphocyte proliferation and maturation (Shin et al., 2017). These promising findings underscore the diverse applications of AD-MSCs and their secretome in regenerative medicine. The varying roles of cytokines, the impact of factors such as MMP-9 and MMP-8 on wound healing, and identifying potential therapeutic targets collectively enrich the landscape of AD-MSC therapy. The intricate interplay of these elements highlights their critical significance in advancing regenerative medicine (Chang and Nguyen, 2021). Croitoru-Lamoury and others explored how the proinflammatory cytokines TNF-α and IFN-γ influence the gene expression of chemokines and their receptors in human mesenchymal stem cells (HuMSCs). HuMSCs were exposed to TNF-α, IFN-γ, or a combination of both for up to 72 h, and gene expression was examined using RT-PCR at various time points (Croitoru-Lamoury et al., 2007). The findings revealed that TNF-α increased the expression of the receptor CXCR4, while both TNF-α and IFN-γ boosted the gene transcription of multiple chemokines (CCL2/MCP-1, CCL3/MIP-1α, CCL4/MIP-1β, CCL5/RANTES, CXCL8/IL-8, CXCL10/IP-10) and cytokines (IL-1β and IL-6). IFN-γ specifically heightened the gene expression of specific chemokines (CXCL9/MIG, CX3CL1/fractalkine) and IL-6. Notably, the combined treatment of TNF-α and IFN-γ synergistically increased the expression of several genes, including CCL3/MIP-1α, CCL4/MIP-1β, CCL5/RANTES, CXCL9/MIG, CXCL10/IP-10, CX3CL1/fractalkine, IL-1β, and IL-6 (Croitoru-Lamoury et al., 2007). One of the most important anti-inflammatory cytokines is IL-10. MSCs can act on macrophages or dendritic cells to produce IL-10, but whether or not MSCs can secret IL-10 by themselves is still controversial (Yagi et al., 2010).

In a very comprehensive and detailed comparative study, it has been demonstrated that MSC from various tissues secreted MCP-1, IL-8, VEGF, IL-6, IL-5, IFNy, and MIP-1β influenced by the age of the culture (Croitoru-Lamoury et al., 2007). In the studies, MSCs from different tissues were tested without treatment, where it was found that fat-derived MSCs secrete the highest levels of IL-6. The pro-inflammatory cytokines TNF-α, IL-2, IL-9, and IL-17, expressed in the supernatant, were associated with myogenic differentiation (Croitoru-Lamoury et al., 2007). The collaborative application of IFN-γ and TNF-α significantly amplifies MSCs’ ability to produce factor H, a pivotal molecule crucial for impeding complement activation (Tu et al., 2010; Saparov et al., 2016). Similar to our result, IL-1b induced TNF-α, IL-6, IL-8, IL-23A, CCL5, CCL20, CXCL10, CXCL11 cytokine secretion along with increased expression of adhesion molecules (VCAM-1, ICAM-1, ICAM-4) (Saparov et al., 2016). When MSC was subjected to multi-cytokine priming involving TNF-α, IL-1β, and IFN-γ, the presence of IL-1β further amplified the well-established immunoregulatory activity initiated by TNF-α/IFN-γ (Hackel et al., 2021). Prolonged treatment of TNFa resulted in similar gene expression and cytokine secretion (IL-4, IL-8, IL6, and IL10 to our findings (Lee et al., 2010; Ting et al., 2021).

However, how these treatments affect the real wound healing ability was not tested. TLR receptors are one of the most ancient components of defense against pathogens and innate immunity. Tests with LPS (TLR4) and PolyI:C (TLR3) showed that MSC lifted their IL-6 and IL-8 expression (Fuenzalida et al., 2016; Park et al., 2016; Vereb et al., 2016; Vereb et al., 2020; Szucs et al., 2023), and the TLR3 manifest a more potent immunosuppressive phenotype (Fuenzalida et al., 2016; Park et al., 2016).

In summary, AD-MSCs have emerged as formidable allies in pursuing effective treatments for non-healing, chronic, and inflamed wounds, even in prolonged inflammation. While these diverse treatments may activate distinct pathways, their cumulative potential in fostering tissue healing, remodeling the extracellular matrix, and promoting regeneration within clinical settings is undeniably remarkable (Hu and Li, 2018; Naji et al., 2019; Pittenger et al., 2019; Mazini et al., 2020; Szucs et al., 2023).

## Data Availability

The original contributions presented in the study are included in the article/Supplementary Material, further inquiries can be directed to the corresponding author.

## Supplementary material

SUPPLEMENTARY FIGURE S1 Successful tri-lineage differentiation of AD-MSCs is demonstrated, with microscopic images illustrating the characteristic features of each tissue type.

SUPPLEMENTARY TABLE S1 The result of Functional Gene Ontology enrichment performed by ViSEAGO.
