# Supplementary figures and images for: Licensing effects of inflammatory factors and TLR ligands on the regenerative capacity of adipose-derived mesenchymal stem cells
Source: Front Cell Dev Biol. 2024 Mar 28;12:1367242. doi: 10.3389/fcell.2024.1367242 (PMC11007080; doi:10.3389/fcell.2024.1367242)

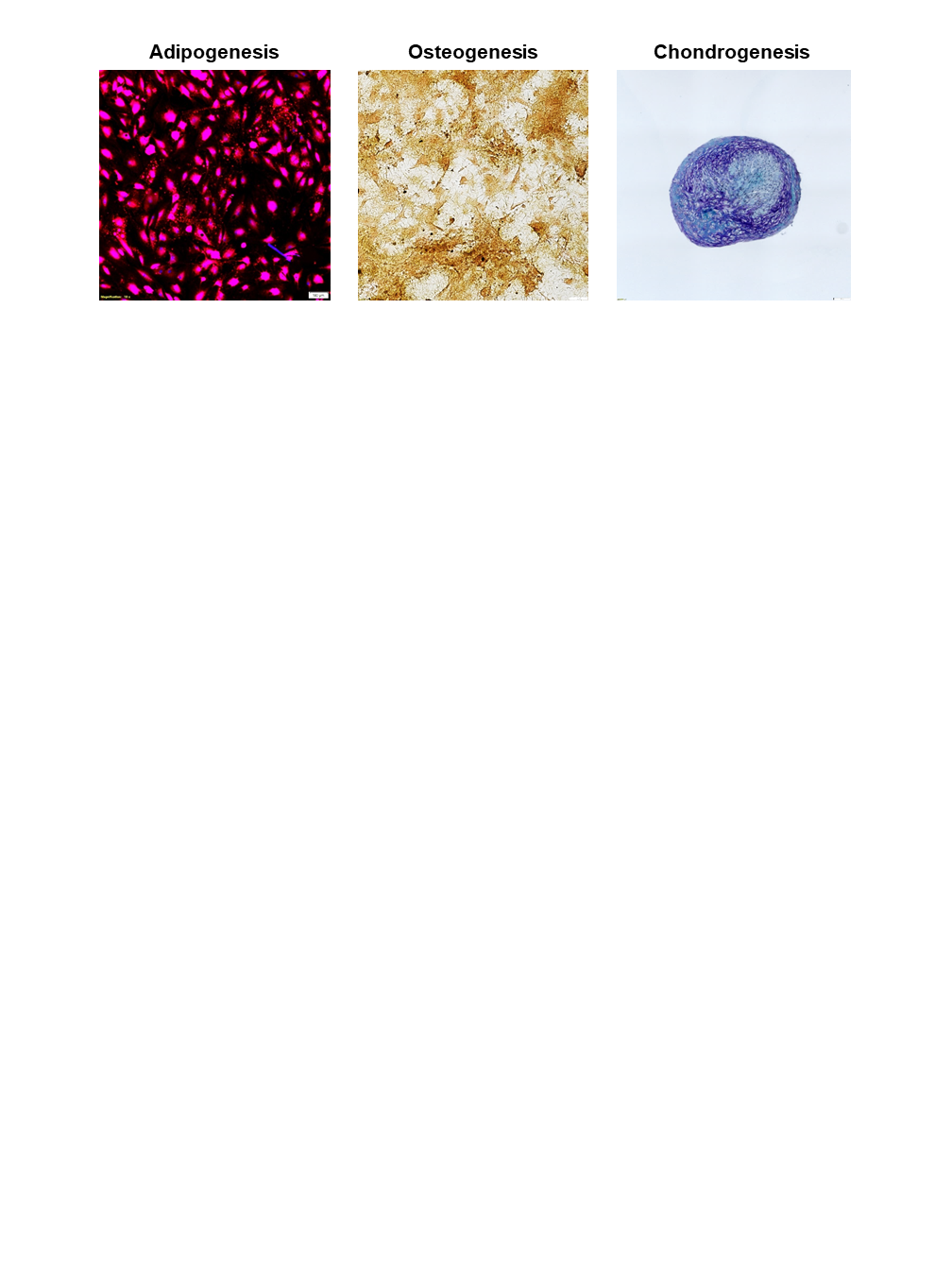

Supplement: Supplementary file 1 [file Image1.TIF]
